# Circulating tumor cell associated white blood cell cluster as a biomarker for metastasis and recurrence in hepatocellular carcinoma

**DOI:** 10.3389/fonc.2022.931140

**Published:** 2022-11-17

**Authors:** Jing Chen, Yuhong Luo, Xiaoxue Xi, Haixia Li, Shufen Li, Lei Zheng, Dinghua Yang, Zhen Cai

**Affiliations:** ^1^ Laboratory Medicine Center, Nanfang Hospital, Southern Medical University, Guangzhou, China; ^2^ Department of Hepatobiliary Surgery, Nanfang Hospital, Southern Medical University, Guangzhou, China

**Keywords:** hepatocellular carcinoma, circulating tumor cell, CTC-WBC cluster, epithelial–mesenchymal transition, cancer metastasis

## Abstract

**Background:**

Recently, an *in vivo* study demonstrated that circulating tumor cell-associated white blood cell (CTC-WBC) cluster possess much greater potential than single CTCs. We aim to explore the correlation between the CTC-WBC cluster and the clinicopathological characteristics of hepatocellular carcinoma (HCC) patients to seek novel biomarkers for HCC metastasis and recurrence.

**Methods:**

We retrospectively analyzed 136 HCC patients from October 2014 to March 2020 who received CTC tests using the CanPatrol CTC enrichment technique. The correlation between the clinical features and total CTCs, EMT-CTCs, and CTC-WBC cluster were analyzed by a chi-square test. The ROC curves were simulated for evaluating the diagnostic performance of CTC parameters in HCC metastasis. Patients were followed up from February 2015 to November 2021, and the relapse-free survival (RFS) was analyzed using the Kaplan–Meier curve.

**Results:**

A total of 93.4% (127/136) and 31.6% (43/136) of HCC patients had detectable CTCs and CTC-WBC clusters. Baseline CTC-WBC cluster was closely correlated with microvascular invasion, portal vein tumor thrombus, and extrahepatic metastasis in pre-treatment HCC patients (*P <*0.05). The simulated ROC curves presented an AUC of 0.821 for the CTC-WBC cluster (sensitivity of 90.0% and specificity of 93.7%) in discriminating metastasis from non-metastatic HCC, which was higher than that for total CTCs (0.718) and EMT-CTCs (0.716). Further follow-up analysis showed that compared to the CTC-WBC cluster negative group (<1/5 ml), patients in the CTC-WBC cluster positive group (≥1/5 ml) presented an increased relapse ratio (60.0% versus 17.9%) and shorter RFS (22.9 versus 53.8 months). Dynamic analysis of CTCs parameters showed that total CTC level, EMT-CTCs proportion, and CTC-WBC cluster were decreased after microwave ablation treatment, while CTC-WBC cluster increased on average 10 months in advance of imaging (MRI) diagnosed recurrence.

**Conclusion:**

The CTC-WBC cluster is a promising biomarker for the metastasis diagnosis and prognosis of HCC metastasis. Dynamic monitoring of the CTC-WBC cluster is an effective method for early detection and intervention of HCC recurrence and metastasis.

## Introduction

Liver cancer is the second leading cause of cancer death in China. An estimated 841,000 newly diagnosed cases of liver cancer and 781,600 deaths occurred worldwide in 2018, with China alone accounting for about 50% of the total number of cases and deaths (1). Most (75%–85%) primary liver cancers are hepatocellular carcinoma (HCC) and are usually not diagnosed until the late stages (1). The high mortality of HCC results from the limited treatment options for advanced-stage HCC and the lack of biomarkers for early detection of cancer metastasis and recurrence.

Circulating tumor cells (CTCs) have been verified to be associated with tumor dissemination and metastasis in many types of cancer, including the common carcinomas of the lung (2), colon (3), breast (4), liver (5), and prostate (6). The prognostic value of CTC enumeration has been recently validated in HCC as well (7, 8). The detection of CTCs from the blood samples of HCC patients is of substantive clinical significance, indicating it might be a potential biomarker for the diagnosis, prognostication, and therapeutic monitoring of HCC (9). It is worth noting that the epithelial–mesenchymal transition (EMT) process, by which polarized epithelial cells lose their adhesion properties and acquire mesenchymal cell phenotypes in CTCs, plays an important role in cancer metastasis. The EMT phenotypes of CTCs are more effective than total CTCs in judging disease progression and prognosis (10–12). It seems that the combined use of EMT-phenotype and total CTCs can enhance the predictive accuracy of assessment of disease progression (5, 13, 14).

A better understanding of CTCs has emerged recently. Szczerba et al. reported that CTCs may interact with immune cells during transmission (15). The interactions between tumor cells and white blood cells (WBCs) could enhance cell cycle progression and lead to more efficient metastasis formation. The CTC-WBC cluster seems to be a new mechanism of tumor invasion and metastasis, and the presence of a CTC-WBC cluster may mean a significantly worse prognosis. A study in breast cancer patients found that patients with a CTC-WBC cluster in peripheral blood had significantly worse overall survival (OS) rates (HR = 7.1, 95% CI: 2.4–21, *P <*0.001) at 6 months (16). In addition, the CTC-WBC cluster was demonstrated to be an independent factor for OS (HR = 2.5, 95% CI: 1.0–6.5, *P <*0.05) in gastric patients (17). Qiu et al. found that positive CTC-WBC cluster patients had significantly shorter disease-free survival (DFS) (HR = 2.0, 95% CI: 1.3–2.8, *P <*0.001) and OS (HR = 3.0, 95% CI: 1.9–4.8, *P <*0.001) than patients with negative CTC-WBC cluster (18).

However, the clinical characteristics of HCC patients with CTC-WBC cluster remain to be clearly defined, and the significance of the CTC-WBC cluster in HCC metastasis and recurrence is not yet fully understood. Moreover, due to the destruction of cell clusters or the concealment of CTC epitopes in the process of CTC enrichment, most CTC isolation equipment may have difficulty in detecting CTC-WBC clusters. Physical separation methods are supposed to be more effective in CTC-WBC cluster detection than biological separation methods since they do not rely on specific markers. In the present study, a novel CanPatrol CTC analysis system (19) was used to isolate CTCs using a nanomembrane filtration method, which enabled the full capture of all types of CTCs and CTC-WBC clusters in peripheral blood. The EMT phenotypes of CTCs were classified by *in situ* hybridizations of RNA fluorescent probes targeting epithelial (EpCAM, CK8, CK18, CK19) and mesenchymal (Vimentin, Twist) markers. All captured CTCs are classified into epithelial CTCs (E-CTCs), hybrid CTCs (H-CTCs), and mesenchymal CTCs (M-CTCs) according to the expression of EMT markers.

Herein, we analyzed the total CTCs, EMT-CTCs, and CTC-WBC clusters in the peripheral blood of HCC patients, aiming to define the characteristics of patients with CTC-WBC clusters and to explore the clinical significance of CTC-WBC clusters in HCC metastasis and recurrence.

## Materials and methods

### Study design

From October 2014 to March 2020, the clinical data of 136 HCC patients treated at the Nanfang Hospital (Guangzhou, China) who received the CanPatrol CTCs test were collected for retrospective analysis. There were 122 males and 14 females, ranging from 25 to 78 years old, with a median age of 50 years. Inclusion criteria: (i) pathological diagnosed as hepatocellular carcinoma; (ii) pathological parameters such as liver cirrhosis, HbsAg, tumor size, TNM stage, CNLC stage, serum AFP level, microvascular invasion (MVI), portal vein tumor thrombus (PVTT), and extrahepatic metastasis were fully recorded; (iii) at least one CTCs and CTC-WBC cluster test results. After admission to the hospital, the patients received surgical resection, interventional or radiofrequency ablation therapy, and necessary follow-up according to the HCC clinical guidelines and physician’s advice. The study was conducted in accordance with the principles of the Declaration of Helsinki, and the study protocol was approved by the Ethics Committee of Nanfang Hospital (NFEC-2018-005).

### CTCs and CTC-WBC cluster analysis

Peripheral blood samples (5 ml, anticoagulated with EDTA) were collected from HCC patients and analyzed by the CanPatrol CTCs detection system (SurExam, Guangzhou, China) before systemic therapy. Besides, blood samples from 20 healthy volunteers were collected to verify the effectiveness of the analytical system. To avoid the affection of physiological differences, we fixed the time and location of blood drawing at 7:00 a.m. to draw blood from the elbow veins of the enrolled individuals. The first 2 ml of blood were discarded before the sample collection to avoid potential skin cell contamination from the venipuncture. The protocols and mRNA probes used for CTC classification were performed as previously described (19). The CTC count, EMT classification, and CTC-WBC cluster were detected by microporous filtration and multiple mRNA *in situ* hybridization technology. Smaller cells, such as single leukocytes, would be removed from filtration. Next, the labeled capture probes targeting CD45 (a marker for leukocytes) and EMT markers were used to indicate specific gene expression. Also, the cells were stained with 4’,6-diamidino-2-phenylindole (DAPI) for the analysis of nuclear morphology. Four fluorescence channels (F1–F4) of the automated imaging microscope were used to detect the signals of different markers (F1-DAPI, F2-CD45, F3-mixed epithelial markers EpCAM/CKs, and F4-mixed mesenchymal markers Vimentin/Twist). Therefore, the large cells with an oval or heteromorphic nucleus and specific chromatin that had no expression of CD45 were identified as CTCs. Next, these CTCs were classified as E-CTCs (red signal), M-CTCs (green signal), and H-CTCs (red and green signals) according to the positive expression of EMT markers. A CTC-WBC cluster was defined as a cluster of cells with white fluorescence (CD45 marker) surrounding a CTC with red, green, or red/green mixed fluorescence signals. The recovery of CTCs was verified previously by spiking 10, 50, 100, and 200 HepG2 cells into 5 ml of blood from healthy volunteers, respectively (19). The average recovery at each dilution of cells ranged from 80% to 89% (R^2^ = 0.999).

### Statistical analyses

SPSS 19.0 (IBM Corp., Armonk, NY, USA) and GraphPad 7.0.0 (GraphPad, San Diego, CA, USA) software were used for statistical analyses. The difference in CTC parameters between groups was analyzed by the Mann–Whitney test. A chi-squared test was used to analyze the relationship between clinicopathological characteristics and CTC parameters. The area under the curve (AUC) of the receiver operating characteristic (ROC) curve was simulated to estimate the diagnostic power of total CTCs, EMT-CTCs, and CTC-WBC clusters in discriminating metastatic HCC patients from non-metastasis patients. The follow-up data were analyzed using the chi-squared test and the Kaplan–Meier curve to compare the relapse ratio and relapse-free survival (RFS). All tests were two-tailed and a P-value under 0.05 was considered significant.

## Results

### Patient characteristics and CTCs analysis

**Figure 1:**
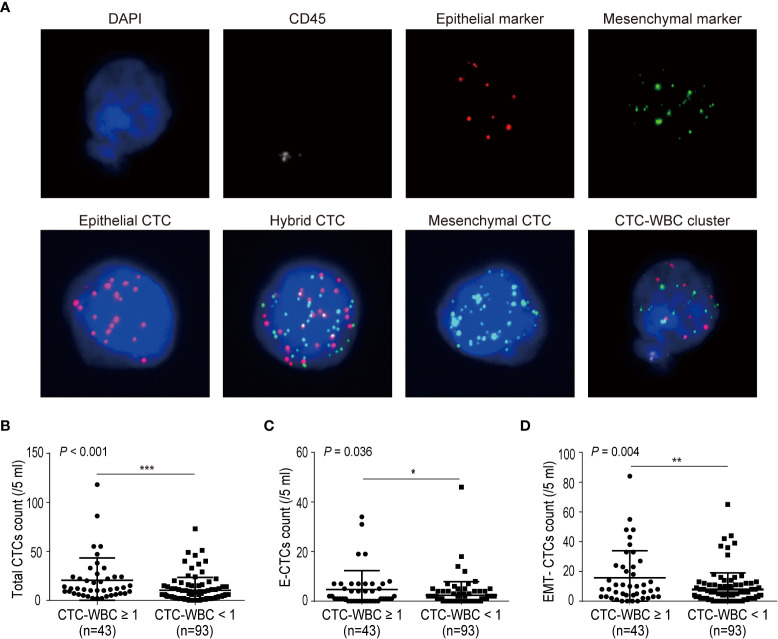
Correlations between the number of CTC-WBC clusters and single CTC parameters (total CTCs, E-CTCs, and EMT-CTCs). **(A)** Images of CTCs (EMT phenotypes) and CTC-WBC cluster. CTCs and CTC-WBC clusters were identified by epithelial and mesenchymal markers. CD45 signal was served as WBC marker and DAPI was used for nuclear staining. **(B–D)** Comparison of total CTCs number **(B)**, E-CTCs number **(C)**, and EMT-CTCs number **(D)** in different CTC-WBC cluster groups (positive or negative). *P <0.05, **P <0.01 and ***P <0.001.

A total of 136 HCC patients were recruited in this study, with a median age of 50 years (range: 25–78 years). Among the patients, 49 patients had MVI, 15 patients had PVTT, and 23 patients were confirmed with metastasis. All clinical details are summarized in **Table 1**. All CTCs were classified by the CanPatrol CTC system through epithelial and mesenchymal RNA probes into three subtypes: E-CTCs (epithelial), H-CTCs (hybrid), and M-CTCs (mesenchymal). A CTC-WBC cluster is defined as a cluster of cells with WBCs surrounding a CTC. The fluorescence images of each CTC subtype and CTC-WBC cluster are shown in **Figure 1A**. To verify the effectiveness of the analytical system, blood samples of 20 healthy volunteers were collected and tested for total CTCs, E-CTCs, M-CTCs, H-CTCs, and CTC-WBC clusters. No CTCs or CTC-WBC clusters were detected in the healthy volunteers, except one donor (No. 16) who presented 1 E-CTC (/5 ml) (**Supplementary Table S1**), for whom further examination and follow-up monitoring was recommended. CTCs were detected (≥1/5 ml) in 127 of 136 (93.4%) HCC patients ranging from 1 to 118 (median: 9)/5 ml. Since CTCs expressing mesenchymal markers were thought to possess stronger metastasis potential than E-CTCs, we divided CTCs into two groups: E-CTCs and EMT-CTCs (including H-CTCs and M-CTCs) based on the expression of EMT makers. The detectable rates of E-CTCs and EMT-CTCs were 61.8% (84/136) and 82.3% (112/136), respectively.

**Table 1:** Clinicopathological characteristics of 136 HCC patients.

Characteristics	Median	Range	N	Percentage (%)
**Gender**	NA^a^	NA		
Male/Female			122/14	89.7/10.3
**Age (years)**	50	25–78		
≥60/<60			30/106	22.1/77.9
**Cirrhosis**	NA	NA		
Yes/No			112/24	82.4/17.6
**HbsAg**	NA	NA		
Positive/Negative			114/22	83.8/16.2
**Tumor size (cm)**	NA	NA		
≥5/<5			69/67	50.7/49.3
**TNM stage**	NA	NA		
I + II/III + IV			88/48	64.7/35.3
**Serum AFP (µg/L)**	9.10	0.0–76,217.0		
≥400/<400			45/91	33.1/66.9
**MVI** ^b^	NA	NA		
Yes/No			49/87	36.0/64.0
**PVTT** ^c^	NA	NA		
Yes/No			15/121	11.0/89.0
**Metastasis**	NA	NA		
Yes/No			23/113	16.9/83.1
**Total CTCs (/5 ml)**	9	0–118		
≥1/<1			127/9	93.4/6.6
**E-CTCs (/5 ml)**	2	0–46		
≥1/<1			84/52	61.8/38.2
**EMT-CTCs (/5 ml)**	8	0–84		
≥1/<1			112/24	82.3/17.7
**CTC-WBC cluster (/5 ml)**	1	0–4		
≥1/<1			43/93	31.6/68.4

a NA, not applicable;

b MVI, microvascular invasion;

c PVTT, portal vein tumor thrombus.

In addition, 43 of 136 (31.6%) patients had detectable CTC-WBC cluster (≥1/5 ml) with a median number of 1 (range: 1–4)/5 ml. We next analyzed the relationship between the number of CTC-WBC clusters and total CTCs or the EMT phenotypes of CTCs (**Figures 1B–D**). The total CTC count was significantly increased in the CTC-WBC cluster ≥1/5 ml group than in the CTC-WBC cluster <1/5 ml group (mean ± SD: 20.4 ± 3.5 vs. 10.3 ± 1.4, *P <*0.001). The E-CTC number was 4.7 ± 1.2 in the CTC-WBC cluster ≥1/5 ml group and 2.4 ± 0.6 in the CTC-WBC cluster <1/5 ml group (*P* = 0.036). The EMT-CTC number was also found to be increased in the CTC-WBC cluster ≥1/5 ml group than in the <1/5 ml group (mean ± SD: 15.5 ± 2.8 vs. 7.8 ± 1.2, *P* = 0.004). These data showed that CTC-WBC cluster number was closely associated with total CTCs count, E-CTCs, and EMT-CTCs number.

### CTC-WBC cluster level correlates to the TNM stage and metastasis in HCC patients

**Figure 2:**
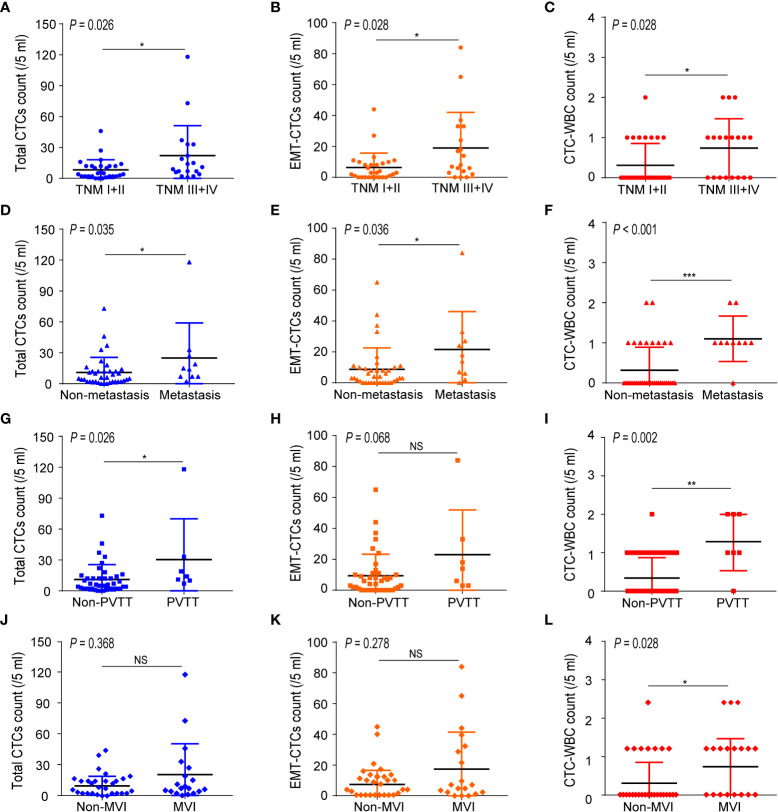
Correlations between the number of CTC parameters (total CTCs, EMT-CTCs, and CTC-WBC cluster) and clinicopathological characteristics of the pre-treated HCC patients. **(A–C)** Comparison of the CTCs parameters between TNM I + II and TNM III + IV groups. **(D–F)** Comparison of the CTCs parameters between non-metastasis and metastasis groups. **(G-I)** Comparison of the CTCs parameters between non-PVTT and PVTT groups. **(J–L)** Comparison of the CTCs parameters between non-MVI and MVI groups. *P <0.05, **P <0.01, ***P <0.001 and NS means not significant.

Next, we analyzed the correlations between baseline CTC parameters (including total CTCs, EMT-CTCs, and CTC-WBC cluster) and the clinicopathological characteristics of 48 pre-treatment HCC patients (**Figure 2**). Compared to the TNM I + II group, the TNM III + IV group presented an increased baseline level of total CTCs (22.1 ± 6.7 vs. 8.4 ± 1.8, *P* = 0.026), EMT-CTCs (19.0 ± 5.3 vs. 6.3 ± 1.7, *P* = 0.028), and CTC-WBC cluster (0.5 ± 0.1 vs. 0.3 ± 0.1, *P* = 0.028) (**Figures 2A–C**). Similarly, compared to the non-metastatic patients, the patients with extrahepatic metastasis showed higher levels of total CTCs (24.9 ± 10.8 vs. 10.9 ± 2.4, *P* = 0.035), EMT-CTCs (21.5 ± 7.8 *vs*. 8.7 ± 2.3, *P* = 0.036), and CTC-WBC cluster (1.1 ± 0.2 vs. 0.3 ± 0.1, *P <*0.001) (**Figures 2D–F**). In the PVTT group, the level of total CTCs (30.3 ± 14.9 vs. 11.0 ± 2.3, *P* = 0.026) and CTC-WBC cluster (1.3 ± 0.3 *vs*. 0.3 ± 0.1, *P* = 0.002) were increased than that in the non-PVTT group; while the difference of EMT-CTCs between the two groups was not significant (23.0 ± 10.9 vs. 9.4 ± 2.2, *P* > 0.05) (**Figures 2G–I**). The level of the CTC-WBC cluster was remarkably higher in the MVI group than that in the non-MVI group (0.7 ± 0.2 vs. 0.3 ± 0.1, *P* = 0.028), whereas the difference of total CTCs (20.5 ± 6.9 vs. 9.5 ± 1.7, *P >*0.05) and EMT-CTCs (17.4 ± 5.5 vs. 7.4 ± 1.7, *P >*0.05) between the two groups was not distinct (**Figures 2J–L**). These data demonstrated that the baseline level of CTC parameters was closely correlated with the TNM stage and extrahepatic metastasis of HCC, and the increased level of baseline CTC-WBC cluster indicated PVTT and MVI status of HCC patients. Besides, there was no significant correlation between the CTC parameters and other clinicopathological characteristics such as age, cirrhosis, HbsAg, tumor size, and serum AFP level (**Supplementary Table S2**).

### CTC-WBC cluster assists in discriminating extrahepatic metastasis in HCC patients

**Figure 3:**
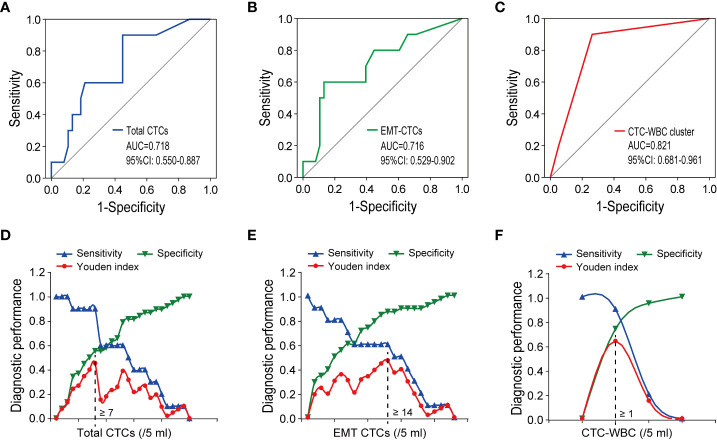
The performance of CTCs parameters (total CTCs, EMT-CTCs, and CTC-WBC cluster) in discriminating the extrahepatic metastatic HCC patients from the non-metastatic patients. **(A–C)** Simulative ROC curves of the CTCs parameters for the diagnosis of HCC extrahepatic metastasis. **(D–F)** Diagnostic performance (sensitivity, specificity, and Youden index) in the discrimination of HCC extrahepatic metastasis.

We further evaluated the ability of total CTCs, EMT-CTCs, and CTC-WBC clusters to diagnose extrahepatic metastasis in 48 pre-treatment HCC patients (10 metastatic and 38 non-metastasis patients) by simulated ROC curves (**Figures 3A–C**). The AUC was 0.718 for total CTCs (95% CI: 0.550–0.887, *P* = 0.035) and 0.716 for EMT-CTCs (95% CI: 0.529–0.902, *P* = 0.037). Compared with them, the CTC-WBC cluster presented better performance with an AUC of 0.821 (95% CI: 0.681–0.961, *P* = 0.002). Based on the ROC curves and Youden index (= sensitivity + specificity − 1), we identified the optimal diagnostic cut-off values for total CTCs, EMT-CTCs, and CTC-WBC clusters in the discrimination of HCC extrahepatic metastasis (**Figures 3D–F**). The optimal cut-off value was 7/5 ml for total CTCs (sensitivity: 90% and specificity: 55.3%) and 14/5 ml for EMT-CTCs (sensitivity: 60% and specificity: 86.8%). The optimal cut-off value of the WBC-CTC cluster was 1/5 ml, presenting more remarkable sensitivity (90.0%) and specificity (93.7%). These data suggest that the CTC-WBC cluster ≥1/5 ml provides a high indication of distant metastasis in HCC patients.

### CTC-WBC cluster indicates cancer recurrence and poor prognosis of HCC patients

**Figure 4:**
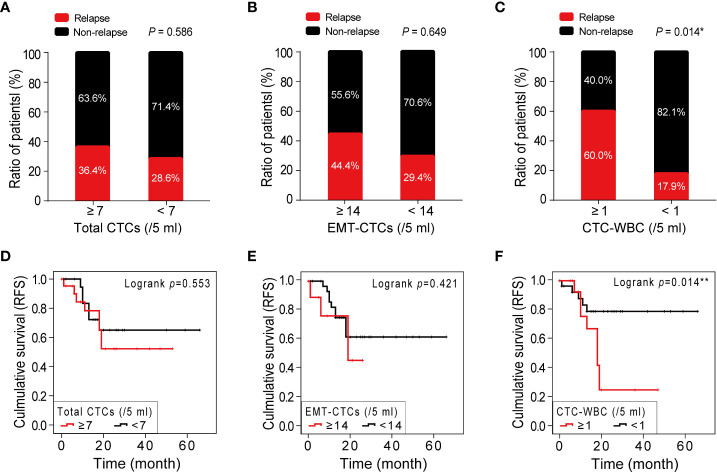
Correlations between the number of CTC parameters (total CTCs, EMT-CTCs, and CTC-WBC cluster) and HCC prognosis. **(A–C)** A comparison of the relapse ratio between the high and low groups of total CTCs **(A)**, EMT-CTCs **(B)**, and CTC-WBC cluster **(C)**. **(D–F)** The Kaplan–Meier survival curves of the high and low groups of total CTCs **(D)**, EMT-CTCs **(E)**, and CTC-WBC cluster **(F)**. *P <0.05 and **P <0.01.

To explore the significance of CTC parameters in the prognosis of HCC, we next analyzed the relationship between the relapse ratio and baseline total CTCs, EMT-CTCs, and CTC-WBC cluster. A total of 43 out of 48 pre-treatment HCC patients were followed-up from February 2015 to November 2021 (range: 2–72 months; mean: 18.0 months) and 34.9% (14/43) had a relapse disease. According to the optimal cut-off values identified by the ROC analysis, patients were divided into CTCs parameter high and low groups to compare the relapse ratio between groups (**Figures 4A–C**). The relapse ratio was 36.4% versus 28.6% (*P >*0.05) in the total CTCs high group (≥7/5 ml) versus low group (<7/5 ml), and 44.4% versus 29.4% (*P >*0.05) in the EMT-CTCs high group (≥14/5 ml) versus low group (<14/5 ml), respectively. However, the relapse ratio in the CTC-WBC cluster positive group (≥1/5 ml) was remarkably higher (60.0% versus 17.9%, *P* = 0.014) than that in the CTC-WBC cluster negative group (<1/5 ml). The Kaplan–Meier survival analysis (**Figures 4D–F**) also showed that patients in the CTC-WBC cluster positive group had a shorter RFS (mean 22.9 months) than those in the CTC-WBC cluster negative group (mean RFS: 53.8 months, *P* = 0.014), whereas there was no significant difference in the RFS between patients in the total CTCs high group versus low group and EMT-CTCs high group versus low group (*P >*0.05). These results revealed that the CTC-WBC cluster could be a prognostic marker for HCC. The baseline increase of the CTC-WBC cluster might indicate relapse risk and poor survival in HCC patients.

### Dynamic monitoring of CTC-WBC cluster in early warning of recurrence in HCC patients

**Figure 5:**
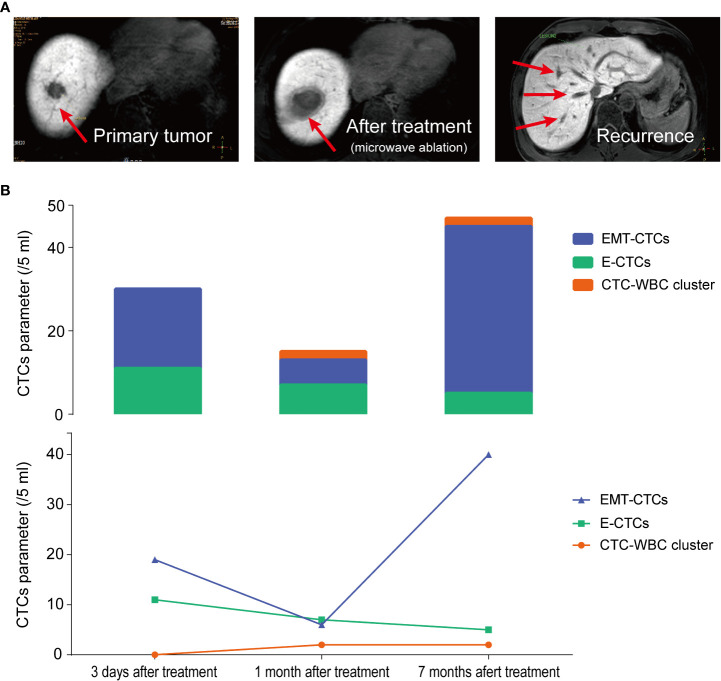
Longitudinal monitoring of total CTC count, EMT-CTC proportion, and CTC-WBC cluster in HCC patient K80570 who received microwave ablation. **(A)** The MRI images of the liver at 3 days, 1 month, and 7 months after microwave ablation treatment are presented. **(B)** Changes of E-CTCs, EMT-CTCs, and CTC-WBC cluster at pre-treatment, 1 month and 7 months after treatment.

According to the therapeutic needs and patient’s intention, 22 patients underwent dynamic CTC monitoring after baseline CTC testing before treatment. The sequential CTC test was conducted 7 days after surgery or after every pharmacotherapy cycle. During subsequent visits, the CTC parameters were monitored every three months. Among 22 HCC patients under dynamic CTC monitoring, 11 patients were diagnosed with recurrence or extrahepatic metastasis during follow-up. Noticeably, in the most recent CTC test, the CTC-WBC cluster was detected in all patients with recurrence (positive rate: 100%) and showed an upward trend at an average of 10 months in advance of imaging (MRI) diagnosed cancer progression. These data further supported that the CTC-WBC cluster was closely related to the metastasis of HCC patients. We selected the most representative one from the longitudinal monitoring of HCC patients and analyzed the dynamic changes of total CTCs, EMT-CTCs, and CTC-WBC clusters during treatment (**Figure 5**). In patient K80570, the total CTC count dramatically decreased 1 month after microwave ablation treatment, and the EMT-CTC proportion also dropped. However, the CTC-WBC cluster was detected with a number of 2/5 ml. Subsequent monitoring found that there was an increased total CTC number and EMT-CTC proportion 7 months after the treatment. The CTC-WBC cluster was also detectable, and MRI-CT finally proved the multi-intrahepatic recurrence. These results indicated the significant value of the post-treatment increase of the CTC-WBC cluster in the early prediction of HCC recurrence.

## Discussion

Studies have shown that CTCs play an important role in tumor recurrence and metastasis (20, 21). Counting and typing of CTCs are considered to be effective biomarkers for predicting the survival and recurrence of HCC and other malignant tumors (7–12). Recently, studies found that CTC-WBC clusters formed by the interaction between CTCs and immune cells expand the metastatic potential of CTCs and lead to more effective metastasis formation than single CTCs (15, 18). However, the clinical significance of the CTC-WBC cluster in HCC metastasis and prognosis remains to be illustrated. In the present study, we analyzed the number of CTC-WBC clusters in the peripheral blood of HCC patients to explore the correlation between CTC-WBC clusters and patient characteristics. The total CTC count and EMT phenotypes of CTCs were simultaneously detected. In addition, longitudinal monitoring of the CTC parameters was conducted to evaluate their significance in the detection of HCC metastasis and recurrence.

The CanPatrol CTC analysis system used in this study is a novel method that enables the detection of CTC-WBC clusters as well as total CTCs and EMT phenotypes of CTCs (18, 22). Using the membrane filtration method that does not rely on surface markers, this system can enrich a variety of CTC subtypes and the CTC-WBC cluster, followed by EMT typing of the enriched CTCs and the CTC-WBC cluster through the fluorescence-labeled epithelial or mesenchymal markers. In the present study, we found a notable increase in total CTCs and EMT-CTCs in the CTC-WBC cluster ≥1/5 ml group compared with that in the CTC-WBC cluster negative group, demonstrating the close correlation between the CTC-WBC cluster and EMT-CTCs in HCC patients. Moreover, most of the CTCs in CTC-WBC clusters detected in our study were EMT type (EMT-CTCs), which was consistent with the previous study in metastatic breast cancer (23), indicating the potential role of CTC-WBC cluster in tumor metastasis.

Further analysis of the pre-treatment HCC patients confirmed that the baseline CTC-WBC cluster was significantly correlated with TNM stage, extrahepatic metastasis, and PVTT, which was in line with a previous study by Luo Q’s team (18). Meanwhile, the total CTCs and EMT-CTCs were also closely related to the TNM stage and extrahepatic metastasis of HCC. The simulated ROC curve presented a higher AUC for the CTC-WBC cluster (0.821) than that for total CTCs (AUC 0.718) and EMT-CTCs (AUC 0.716) in discriminating HCC patients with extrahepatic metastasis from non-metastatic patients. Baseline increase of CTC-WBC cluster (≥1/5 ml) could provide an indication of distant metastasis in HCC patients, with a sensitivity of 90.0% and specificity of 93.7%. These results highlight the diagnostic performance of the CTC-WBC cluster in HCC extrahepatic metastasis. The intrinsic mechanisms of the greater potential of the CTC-WBC cluster in promoting cancer metastasis might be related to the enhanced cell proliferation and immune escape of the WBC combined CTCs. Szczerba et al. used single-cell RNA sequencing to analyze the gene profiles of cells in CTC-WBC clusters from breast cancer patients (15). They found that most CTCs involved in CTC-WBC clusters were neutrophils, and several genes that modulate cell cycle progression were identified as differentially expressed in CTCs associated with neutrophils compared with those of CTCs alone, thus leading to more efficient metastasis formation. The research of Wang et al. also found that the number of CTC-WBC clusters in HCC patients was related to a specific genome profile involving cell adhesion and cell movement, which may contribute to the metastasis process (24). Fundamental research on prostate cancer demonstrated that neutrophils could bind to CTCs through intercellular adhesion molecule-1 to enhance the immune-killing resistance of CTCs to NK cells, thereby promoting the immune escape of CTCs (25). However, further investigations are needed to illustrate the interaction between CTCs and WBCs and the molecular mechanisms involved in promoting HCC metastasis.

What is noteworthy is that we also found a remarkable increase in CTC-WBC cluster number in the MVI group compared with the non-MVI group, whereas the difference in total CTCs and EMT-CTCs between the two groups was not significant. MVI refers to the nests of cancer cells in the small branches of the portal vein, hepatic vein, or hepatic artery that are only visible under the microscope (no large vessel tumor thrombus) (26). As a biomarker of early metastasis in HCC, MVI is the most important risk factor for the recurrence of HCC after resection treatment (27, 28). Further follow-up analysis confirmed that compared to the CTC-WBC cluster negative group (<1/5 ml), patients in the CTC-WBC cluster positive group (≥1/5 ml) presented an increased relapse ratio (60.0% versus 17.9%) and shorter RFS (22.9 versus 53.8 months). These data suggest that the CTC-WBC cluster might be a sensitive prognostic marker for HCC patients, which is consistent with the previous study that demonstrated the CTC-WBC cluster was an independent prognostic indicator of DFS (HR = 2.0, 95% CI: 1.3–2.8) and OS (HR = 3.0, 95% CI: 1.9–4.8) in HCC patients (18). Furthermore, dynamic changes of the CTC-WBC cluster in the longitudinal monitoring of HCC patients showed that the detection rate of the CTC-WBC cluster was 100% in patients with recurrence, and the CTC-WBC cluster had appeared 10 months in advance of the MRI imaging diagnosed HCC recurrence. Therefore, dynamic monitoring of CTCs after treatment, especially the dynamic changes of the CTC-WBC cluster, is expected to be an important method for predicting the extremely early recurrence of HCC patients and provide valuable clues for early intervention and treatment of HCC recurrence and metastasis.

In summary, this work demonstrated the significant value of the CTC-WBC cluster in HCC metastasis and recurrence. The CTC-WBC cluster could be a promising biomarker for the diagnosis of metastasis and prognosis of HCC. Meanwhile the dynamic change of the CTC-WBC cluster provides vital clues for early detection and intervention of HCC recurrence. The limitation of this study is that the enrolled sample is limited. More exploration and verification are needed before this newly developed liquid biopsy biomarker can be used in clinical practice, though the findings of the present study are encouraging. In the future, an expanded sample size and various tumor types are planned to further verify the clinical significance of the CTC-WBC cluster in HCC and other malignant cancers. Since the development of novel technology for CTC analysis is growing very fast, multicenter investigations are expected to solve the lack of a unified technical standard for CTC tests. This will enable us to have suitable cutoff values for CTC parameters in different clinical scenarios such as diagnosis, medication guidance, and prognosis. In addition, the exact classification of the CTC-WBC clusters using probes targeting specific markers (such as CD11b and Gr-1 marker for neutrophils) and *in vitro* and *in vivo* experimental investigations will be conducted to explore their function and molecular mechanism in promoting tumor metastasis.

## Data availability statement

The original contributions presented in the study are included in the article/**Supplementary Material**. Further inquiries can be directed to the corresponding authors.

## Ethics statement

The studies involving human participants were reviewed and approved by the Ethics Committee of Nanfang Hospital, Southern Medical University. Written informed consent for participation was not required for this study in accordance with the national legislation and the institutional requirements.

## Author contributions

JC, DY, and ZC conceived the research. JC, YL, XX, and HL collected the clinical data of patients. YL, XX, and SL conducted the statistical analysis. JC, XX, LZ, and ZC handled the manuscript editing and review. All authors listed have made a substantial, direct, and intellectual contribution to the work and approved it for publication.

## Funding

This work was funded by the Science and Technology Planning Project of Guangdong Province of China (2016A010105006), the President Foundation of Nanfang Hospital, Southern Medical University (2019A002, 2020C001), and the National Natural Science Foundation of China (82102493).

## Conflict of interest

The authors declare that the research was conducted in the absence of any commercial or financial relationships that could be construed as a potential conflict of interest.

## Publisher’s note

All claims expressed in this article are solely those of the authors and do not necessarily represent those of their affiliated organizations, or those of the publisher, the editors and the reviewers. Any product that may be evaluated in this article, or claim that may be made by its manufacturer, is not guaranteed or endorsed by the publisher.
