# Supplementary material for: Circulating tumor cell associated white blood cell cluster as a biomarker for metastasis and recurrence in hepatocellular carcinoma
Source: Front Oncol. 2022 Nov 17;12:931140. doi: 10.3389/fonc.2022.931140 (PMC9713305; doi:10.3389/fonc.2022.931140)
Supplement: Supplementary file 1 [file DataSheet_1.docx]

Supplementary Material

**Table S1** CTCs test (total CTCs, E/H/M-CTCs and CTC-WBC clusters) of healthy donors (n=20)

| **No.** | **Total CTCs** | **E-CTCs** | **H-CTCs** | **M-CTCs** | **CTC-WBC clusters** |
| --- | --- | --- | --- | --- | --- |
| 1 | 0 | 0 | 0 | 0 | 0 |
| 2 | 0 | 0 | 0 | 0 | 0 |
| 3 | 0 | 0 | 0 | 0 | 0 |
| 4 | 0 | 0 | 0 | 0 | 0 |
| 5 | 0 | 0 | 0 | 0 | 0 |
| 6 | 0 | 0 | 0 | 0 | 0 |
| 7 | 0 | 0 | 0 | 0 | 0 |
| 8 | 0 | 0 | 0 | 0 | 0 |
| 9 | 0 | 0 | 0 | 0 | 0 |
| 10 | 0 | 0 | 0 | 0 | 0 |
| 11 | 0 | 0 | 0 | 0 | 0 |
| 12 | 0 | 0 | 0 | 0 | 0 |
| 13 | 0 | 0 | 0 | 0 | 0 |
| 14 | 0 | 0 | 0 | 0 | 0 |
| 15 | 0 | 0 | 0 | 0 | 0 |
| 16 | 1 | 1 | 0 | 0 | 0 |
| 17 | 0 | 0 | 0 | 0 | 0 |
| 18 | 0 | 0 | 0 | 0 | 0 |
| 19 | 0 | 0 | 0 | 0 | 0 |
| 20 | 0 | 0 | 0 | 0 | 0 |

**Table S2** Association between CTC-WBC clusters and clinical features in pre-treated HCC patients

| **Groups** | **Total CTCs** | | | | **EMT-CTCs** | | | | **CTC-WBC clusters** | | | |  |
| --- | --- | --- | --- | --- | --- | --- | --- | --- | --- | --- | --- | --- | --- |
|  | ≥ 9 | < 9 | χ^2^ | *P* | ≥ 8 | < 8 | χ^2^ | *P* | ≥ 1 | < 1 | χ^2^ | *P* | |
| **Gender** |  |  | 0.489 | 0.484 |  |  | 0.215 | 0.643 |  |  | 2.091 | 0.148 | |
| Male | 21 | 20 |  |  | 19 | 22 |  |  | 14 | 27 |  |  | |
| Female | 2 | 5 |  |  | 2 | 5 |  |  | 5 | 2 |  |  | |
| **Age (years)** |  |  | 0.362 | 0.548 |  |  | 1.148 | 0.284 |  |  | 2.433 | 0.119 | |
| ≥ 60 | 3 | 6 |  |  | 2 | 7 |  |  | 1 | 8 |  |  | |
| < 60 | 20 | 19 |  |  | 19 | 20 |  |  | 18 | 21 |  |  | |
| **Cirrhosis** |  |  | 0.362 | 0.548 |  |  | 0.106 | 0.744 |  |  | 0.646 | 0.422 | |
| Yes | 20 | 19 |  |  | 18 | 21 |  |  | 17 | 22 |  |  | |
| No | 3 | 6 |  |  | 3 | 6 |  |  | 2 | 7 |  |  | |
| **HbsAg** |  |  | 0.107 | 0.743 |  |  | 0.012 | 0.912 |  |  | 0.012 | 0.911 | |
| Positive | 21 | 21 |  |  | 19 | 23 |  |  | 16 | 26 |  |  | |
| Negative | 2 | 4 |  |  | 2 | 4 |  |  | 3 | 3 |  |  | |
| **Tumor size** |  |  | 0.426 | 0.514 |  |  | 0.035 | 0.853 |  |  | 2.315 | 0.128 | |
| ≥ 5 cm | 15 | 14 |  |  | 13 | 16 |  |  | 14 | 15 |  |  | |
| < 5 cm | 8 | 11 |  |  | 8 | 11 |  |  | 4 | 14 |  |  | |
| **AFP (µg/L)** |  |  | 0.251 | 0.616 |  |  | 0.042 | 0.838 |  |  | 3.594 | 0.058 | |
| ≥ 400 | 7 | 6 |  |  | 6 | 7 |  |  | 8 | 5 |  |  | |
| < 400 | 16 | 19 |  |  | 15 | 20 |  |  | 11 | 24 |  |  | |
